# A Gini approach to spatial CO_2_ emissions

**DOI:** 10.1371/journal.pone.0242479

**Published:** 2020-11-18

**Authors:** Bin Zhou, Stephan Thies, Ramana Gudipudi, Matthias K. B. Lüdeke, Jürgen P. Kropp, Diego Rybski

**Affiliations:** 1 Potsdam Institute for Climate Impact Research, Member of the Leibniz Association, Potsdam, Germany; 2 Jacob Blaustein Institutes for Desert Research, Ben-Gurion University of the Negev, Beersheba, Israel; 3 Department of Geo- and Environmental Sciences, University of Potsdam, Potsdam, Germany; 4 Department of Environmental Science Policy and Management, University of California Berkeley, Berkeley, CA, United States of America; Institute for Advanced Sustainability Studies, GERMANY

## Abstract

Combining global gridded population and fossil fuel based CO_2_ emission data at 1 km scale, we investigate the spatial origin of CO_2_ emissions in relation to the population distribution within countries. We depict the correlations between these two datasets by a quasi-Lorenz curve which enables us to discern the individual contributions of densely and sparsely populated regions to the national CO_2_ emissions. We observe pronounced country-specific characteristics and quantify them using an indicator resembling the Gini-index. As demonstrated by a robustness test, the Gini-index for each country arise from a compound distribution between the population and emissions which differs among countries. Relating these indices with the degree of socio-economic development measured by per capita Gross Domestic Product (GDP) at purchase power parity, we find a strong negative correlation between the two quantities with a Pearson correlation coefficient of -0.71. More specifically, this implies that in developing countries locations with large population tend to emit relatively more CO_2_, and in developed countries the opposite tends to be the case. Based on the relation to urban scaling, we discuss the implications for CO_2_ emissions from cities. Our results show that general statements with regard to the (in)efficiency of large cities should be avoided as it is subject to the socio-economic development of respective countries. Concerning the political relevance, our results suggest a differentiated spatial prioritization in deploying climate change mitigation measures in cities for developed and developing countries.

## Introduction

Urbanization is an ongoing process in many parts on the globe. It is projected that due to rural-urban migration much of the future urbanization is going to take place in developing and transition countries. This leads to ever more mega-cities [1, 2]. In parallel, humanity is facing another challenge, namely climate change. To date, cities, despite occupying less than 1% of the global land area, account for more than 70% of the anthropogenic green house gas (GHG) emissions [3]. Therefore, cities are often identified as the key focal areas for global mitigation actions. While a large contribution of the global CO_2_ emissions is commonly attributed to cities [4], the CO_2_ reduction role of further urbanization is also discussed with the argument of efficiency gains associated with the high densities in cities [5]. Moreover, cities are known to perform more efficiently in addressing the basic needs of human beings [5]. Hence, a diversified view on cities is needed and in view of climate change mitigation, a better understanding of the interplay between urbanization, origin of CO_2_ emissions, and socio-economic development is of great interest.

Globally, cities are characterized by higher population densities compared to rural areas. Recent literature has identified the crucial role played by population density in either increasing or decreasing the emission efficiency in cities [6–10]. The impact of population density on reducing/increasing CO_2_ emissions in these studies is either calculated based on specific assumptions made to calculate the city specific CO_2_ emissions or through the construction of city clusters using a clustering algorithm, see [11, 12]. However, most of these studies are limited to a specific country or a region. Therefore, there is a gap in the existing literature about the sub-national origin of CO_2_ emissions at a global scale. Bridging this gap would provide better insights as to whether population density is a crucial factor in improving/decreasing emission efficiency and would identify other factors that influence CO_2_ emissions at a country scale.

Here, we investigate how the spatial origin of CO_2_ emissions relates to the spatial distribution of population. We address the questions, to which extent locations of large population also emit the most CO_2_ and if there is any dependence on human development. In order to avoid discussions about the proper city definition, the correlations are analyzed on the level of grid cells—keeping in mind that locations of high population are likely to belong to cities. Thus, we analyze population and CO_2_ emissions by employing a quasi-Lorenz curve that relates the cumulative population and cumulative emissions for entire countries on a grid-cell level (the Lorenz curve was originally used to describe unequal income distribution).

The shape of these curves explains whether the emissions are concentrated in locations of high or low population. Inspired by the apparent similarity, we extend the well-known Gini-index. Based on the data employed, we find that within many countries, locations with high or low population exhibit different relative emissions. We thus compare the extended “Gini-index” with the economic strength of the considered countries (as captured by the GDP per capita) which can be to some extent interpreted as a measure for the stage of development. We further hypothesize that the development stage of respective countries plays an important role in explaining this relationship.

Earlier studies attempted to address the emission efficiency of densely populated regions by means of urban scaling, where an urban indicator is plotted against the city size in terms of population [13]. The exponent, estimated as the slope of a linear regression in the log-log representation, quantifies efficiency gains of large or small cities. However, in case of urban CO_2_ emissions, published results from urban scaling leave an inconclusive picture (for an overview we refer to [14, 15]). In the present work we address this issue by combining high resolution, global population and CO_2_ emission data sets in order to quantify whether locations with high or low population emit more or less CO_2_. We further discuss an analytic link between our approach and urban scaling.

## Materials and methods

### Population data

**Fig 1:**
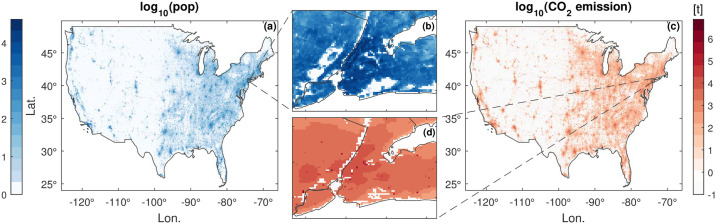
Population and CO_2_ emissions for the contiguous USA. (a) Gridded Population of the World, version 4, GPWv4 [17] at 1 × 1 km^2^ spatial resolution in 2010 and (c) total anthropogenic CO_2_ emission from ODIAC data [18] for the same region, year, and resolution. (b) and (d) depict magnified views of population and CO_2_ emission for the New York metropolitan region, respectively. Visually, large agglomerations of population coincide with large amounts of emissions. To which extent they relate proportionally is the subject of this paper.

We used the Gridded Population of the World, version 4 (GPWv4) population count data for the year 2010 [16]. GPWv4 data allocate the population counts of census units collected globally from various institutions into standard 1 × 1 km^2^ grid cells by means of an areal-weighting interpolation [17]. Fig 1(a) illustrates the GPWv4 data in the year 2010 for the contiguous US. The distribution of population in the US exhibits an inhomogeneity. The metropolitan urban agglomerations accommodate a large share of population in the US, whereas the states in the Mountain West are generally sparsely populated.

### CO_2_ emissions data

Fossil fuel based CO_2_ emission estimates are obtained from the Open source Data Inventory of Anthropogenic CO_2_ (ODIAC) emissions of version ODIAC2015a available globally at 1 × 1 km^2^ grid for the year 2010 [18]. In the ODIAC dataset, point sources, i.e. power plant emissions obtained from the database CARMA (Carbon Monitoring and Action) are directly assigned to the grids, while non-point sources (e.g. emissions from transport, industrial, residential, and commercial sectors) are disaggregated based on global and national emission estimates made by the Carbon Dioxide Information Analysis Center (CDIAC) [19], using remotely sensed nightlight data as a proxy. An exception is the emissions from cement production which have point source origins but are spatially disaggregated as non-point sources. Non-land emissions, such as those from international bunkers (international aviation and maritime shipping), are assigned to the non-point emissions.

Compared with conventional population-based approaches, the nightlight data can trace the human activities more appropriately [20, 21]. Worthy of special mention is that the gridded emission data of ODIAC used in this study is not disaggregated using population density as a proxy. Therefore, the two datasets depict distinguishing zonal patterns, as shown in the example for the New York metropolitan region in Fig 1(b) and 1(d). Without relying on the time-consuming update of census data, emissions allocated using nightlights can be updated more frequently and may be of particular importance for developing countries where conducting census is still a challenge. Fig 1(c) shows the gridded total anthropogenic CO_2_ emissions (in tons) for the year 2010 for the contiguous US, analogous to the population data shown in Fig 1(a). As observed, the emissions also exhibit pronounced inhomogeneities.

In order to check the consistency of the results obtained, we further compare our results obtained from the ODIAC data with other CO_2_ emission datasets, namely the Fossil Fuel Data Assimilation System (FFDAS) version 2.0 [22] and the Emission Database for Global Atmospheric Research (EDGAR) version 4.3.2 [23]. Both data are for the year 2010. For the sub-national analysis we also analyze the Vulcan data, which has been analyzed before [12]. However, we focus on ODIAC, since it has the highest resolution, and we discuss the results of other datasets in comparison to the ODIAC results.

The fundamentals of creating the four gridded CO_2_ emission inventories used in this study have been compared and discussed in detail in [24]. In general, they differentiate themselves in terms of 1) the energy statistics used which determines the sectors included in calculating the total national CO_2_ emissions, and 2) the approach to disaggregating and allocating the CO_2_ emissions to a regular grid.

Dissimilarities among the inventories may be dominated by the disaggreation method. FFDAS applies the Kaya identity to balance CO_2_ emissions across regions, relying on population and nightlight data [22, 25] (see also [15] for further information on the Kaya identity). Viewed as the most accurate emissions inventory, a bottom-up method has been used for the Vulcan data allocate large point sources, road-specific emissions, and non-point emissions to census tracts, and further resampled to a 10-km grid [26]. However, since the subnational emissions data are not always available, the Vulcan data is restricted to the USA at the moment.

### Inhomogeneity index of CO_2_ emissions *G_e_*

In order to characterize the relation between country-wise population and CO_2_ emissions, we plotted the cumulative quantities against each other. We sorted the grid cells of a country by population in ascending order and calculated the cumulative share of population and CO_2_ emissions arising therefrom. Then we interpreted the cumulative emissions as a function of the corresponding cumulative population.

The plotted curves resemble the so-called *concentration curves* used to describe socio-economic inequalities. The most popular concentration curve is the Lorenz curve usually employed to visualize income inequalities. Other applications of concentration curves include for example the analysis of socio-economic inequalities in the health sector (e.g. [27]). Since the curves we compute here, do not agree exactly with the classical definition of a concentration curve we will refer to them as *quasi-Lorenz curves*. We justify the choice of this method by its simplicity—it only requires sorting—and the fact that it does not require any parameters or assumptions on functional forms.

**Fig 2:**
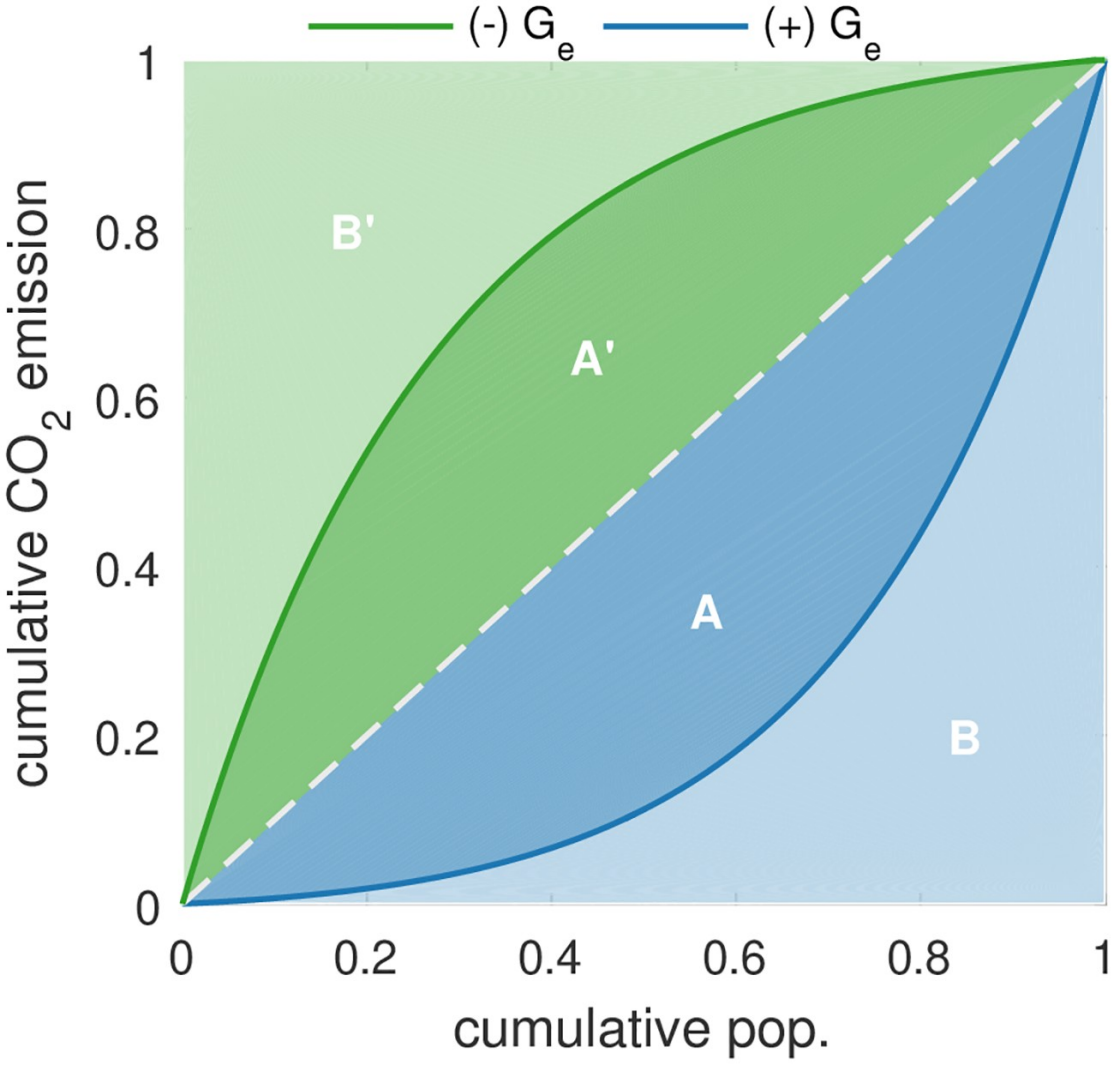
Illustration of the inhomogeneity index *G_e_*. Quasi-Lorenz curves (solid lines) and the calculation of *G_e_*, which is inspired by the Gini coefficient: *G_e_*_+_ = *A*/(*A* + *B*) and *G_e_*_−_ = −*A*′/(*A*′ + *B*′).

To quantify the curves, we break the shape of each curve down to a single number. As it is well known and used in this context, we generalize the Gini coefficient, which originally has been introduced to quantify income inequality [28]. As illustrated in Fig 2, we distinguish between curves above or below the dashed line with a slope of 45°—the line of equality. For the blue quasi-Lorenz curve, we defined an inhomogeneity index as the ratio of the area between the curve and the line of equality (marked as *A*) to the total area below the line of equality (*A* + *B*). Analogously, the inhomogeneity index of the green curve is −*A*′/(*A*′ + *B*′). We arbitrarily assign the inhomogeneity index for the curves above the line of equality negative, and below positive.

## Results

### Inhomogeneity of emissions across countries

**Fig 3:**
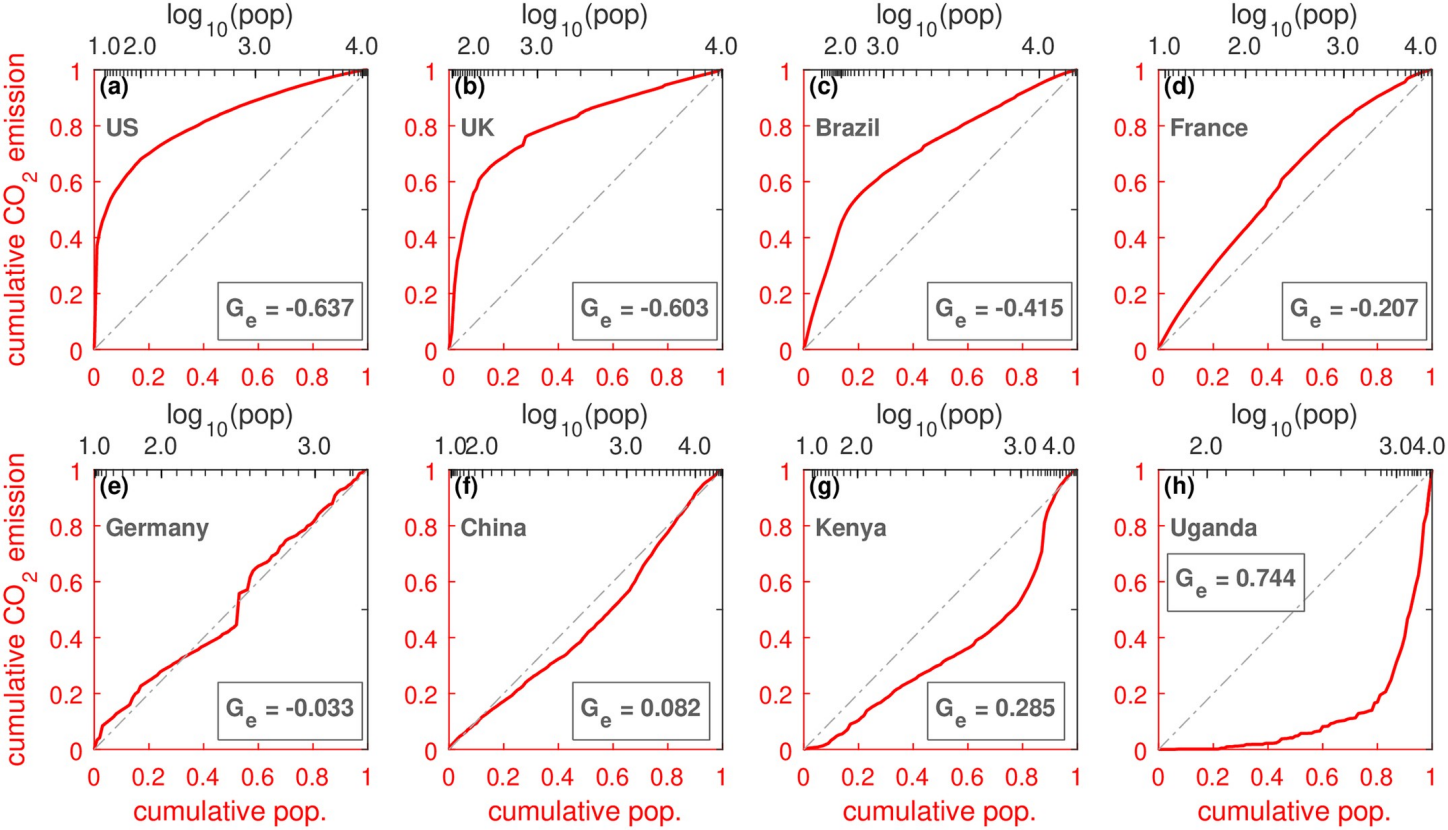
Quasi-Lorenz curves and corresponding inhomogeneity index *G_e_* for selected countries. The country-specific curves are drawn by plotting the accumulated population (in ascending order) on the horizontal axis against the accumulated share of CO_2_ emissions of the corresponding grid cells. The panels show the curves for (a) USA, (b) UK, (c) Brazil, (d) France, (e) Germany, (f) China, (g) Kenya, and (h) Uganda. If the curves follow the diagonal, then low and high densities have the same emissions per capita. If the curves are bent to the lower right corner, then cells of small density exhibit relatively low emissions and high population cells exhibit relatively high emissions. Curves in the upper left corner indicate the opposite behavior. The inhomogeneity index *G_e_* is positive or negative. It can be seen, that various countries exhibit non-proportional relations between population and emissions. The inhomogeneity index *G_e_* seems to be related to the development of the country.

Fig 3 shows the quasi-Lorenz curves for a few countries. The slopes of the curves depend on per capita emissions. In the case of constant emissions per capita, the cumulative share of population and CO_2_ emissions are proportional to each other and follow the diagonal. This is approximately the case for Germany, Fig 3(e). In the case of the USA, Fig 3(a), and the UK, Fig 3(b), the curves are bent to the upper left corner, i.e. the grid cells with small population already include a large amount of emissions (large slope). On the contrary, the curves, e.g., for Uganda, Fig 3(h), and Kenya, Fig 3(g), are bent to the lower right corner. Many grid cells with small population are necessary to include a fair amount of emissions (small slope). Accordingly, curves bent to upper left indicate high per capita emissions in sparsely populated cells and comparably lower per capita emissions in densely populated cells, and vice versa.

In Germany, per capita CO_2_ emissions of large cities are smaller than those of small ones, but the difference seems to be minor [29]. In contrast, per capita CO_2_ in the UK emissions remarkably diverge between large and small cities, ranging from 25.6 tonnes per capita in Middlesbrough to 5.4 tonnes per capita in London in 2012, reflecting the impact of industrial base [30, 31].

Interestingly, in Fig 3 developed countries seem to belong to the group where the curves extend to the upper left corner and less developed countries seem to belong to the group where the curves extend to the opposite corner.

### *G_e_* versus GDP per capita at trans-national level

In order to verify whether there is a systematic relationship between the curve type and the level of countries’ economic development, we plotted the values of the inhomogeneity index *G_e_* for a large number of countries against the logarithm of GDP Purchasing Power Parity (PPP) per capita obtained from the World Bank, an important indicator for economic development.

**Fig 4:**
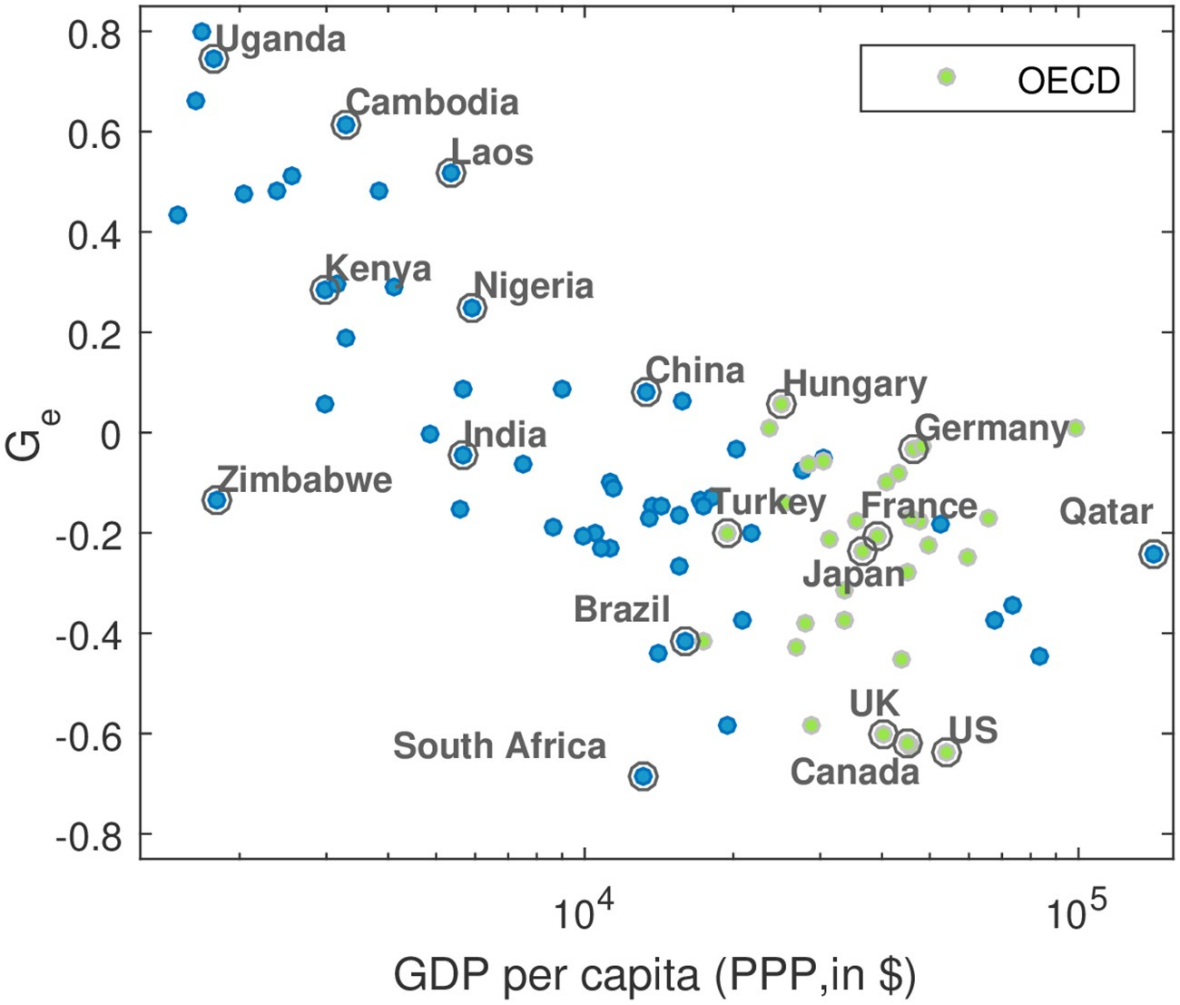
Development dependence of CO_2_-population-inhomgeneity. The inhomogeneity index *G_e_* is plotted vs. Gross Domestic Product (GDP) per capita (PPP) for 94 countries on a semi-logarithmic scale. For better readability only the symbols of a sub-set of countries are labeled. As can be seen, the *G_e_* correlate with the economic development. The Pearson correlation coefficient between *G_e_* and GDP on a logarithmic scale is *ρ* = −0.71 (*p* ≤ 0.01). In more developed countries high population densities have lower emissions as low densities. The GDP data were obtained from the World Bank (http://data.worldbank.org), measured in USD of the year 2010.

As observed in Fig 4, the two quantities correlate (with a Pearson correlation coefficient *ρ* = −0.71, *p* ≤ 0.01). In general, for developed countries *G_e_* tends to have smaller values, and for developing ones it tends to have larger values. Thus, we generalize that in economically developing countries, high population densities are more emission intense and the opposite is the case in economically developed countries.

We repeated the analysis for the FFDAS and the EDGAR data. For reasons of consistency, we also analyze the ODIAC data aggregated to 10 × 10 km^2^ resolution. For the three datasets, the resulting *G_e_*-values are plotted against the GPD per capita, analogous to Fig 4.

**Fig 5:**
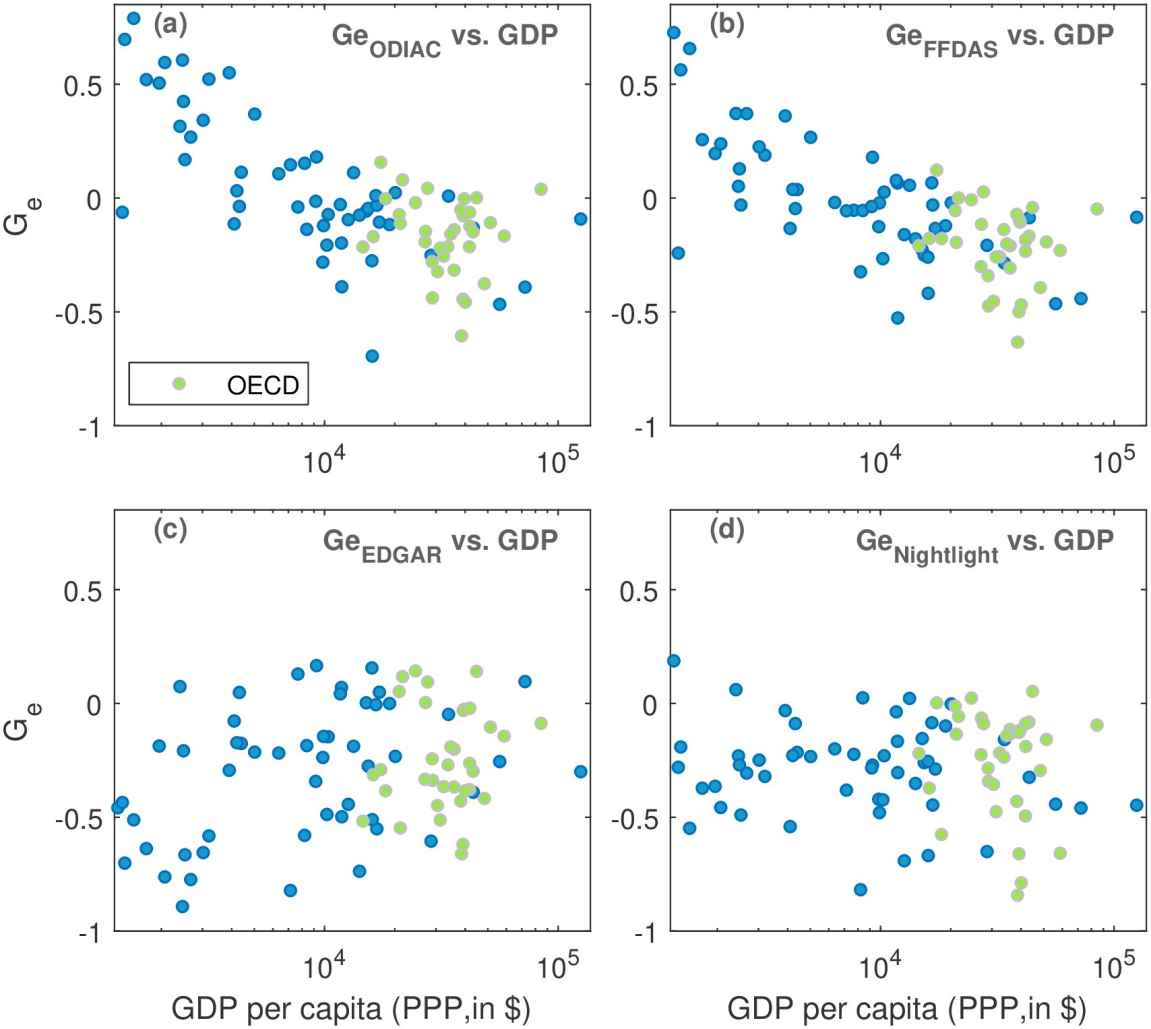
Comparison of CO_2_-population-inhomgeneity for different CO_2_ datasets and nightlights. (a) ODIAC, (b) FFDAS, (c) EDGAR, (d) nightlights [32]. Each panel is analogous to Fig 4, but for consistency of spatial resolution the underlying ODIAC data in (a) has been aggregated to 10 km resolution.

Fig 5(b) reveals a very similar development dependence when FFDAS data are applied as for ODIAC [Fig 5(a)]. In contrast, for the EDGAR data [Fig 5(c)] the development dependence vanishes and is even slightly inverted (*ρ* = 0.29, *p* ≤ 0.01). Differences between the *G_e_*-values of the EDGAR and ODIAC or FFDAS data are most pronounced for developing countries. The difference in these results could be attributed to the poor quality of population census, high demographic dynamics, and insufficient geo-spatial data in developing countries. However, further investigation is needed in order to understand which of these methodological differences factor more with regard to the pronounced dissimilarities in the results. EDGAR relies on road networks, population density and agriculture land use data to downscale the national emissions, which renders it more sensible to errors embedded in the proxy datasets. In comparison, FFDAS uses, besides population density, nightlight data to disaggregate emissions.

Moreover, since ODIAC and FFDAS are at least partly based on nightlight data for the subnational disaggregation [25, 33, 34], one may argue that the development dependence in Fig 5(a) and 5(b) are simply due to such an effect in the nightlight data. Thus, we also analyzed the nightlight data from the Visible Infrared Imaging Radiometer Suite (VIIRS) Day/Night Band (DNB) data [32] in an analogous way as the emissions data and the results are displayed in Fig 5(d). As can be seen, for nightlight data, we do not see any correlations between *G_e_*-values and the GDP per capita. Accordingly, we conclude that the development dependence found in the ODIAC and FFDAS data is not stemming from the nightlight data. Overall, *G_e_*-values tend to be negative for nightlights, indicating that locations of low population have a relatively strong contribution.

### *G_e_* versus GDP per capita at sub-national level

**Fig 6:**
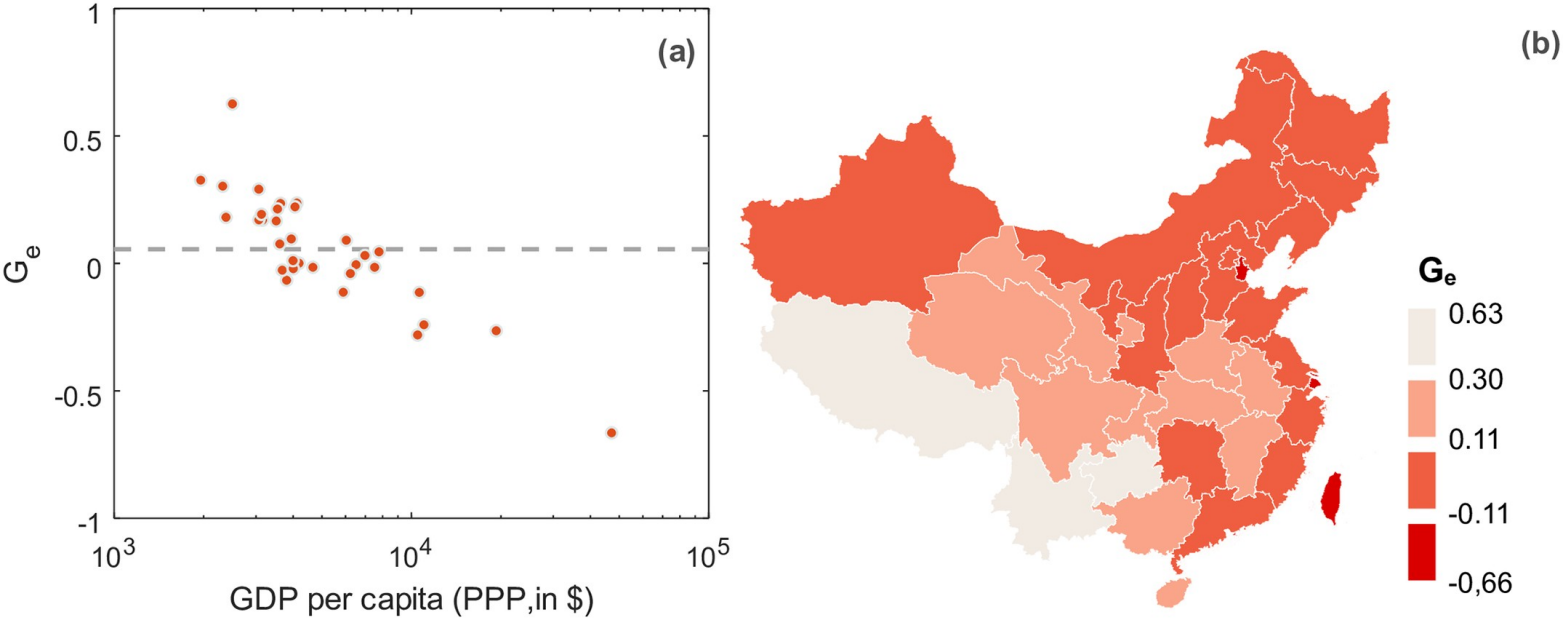
Sub-national inhomogeneity index *G_e_*. We calculated the *G_e_* on the province level for China. In (a) the *G_e_*-values are plotted against the corresponding province GDP per capita values on a logarithmic scale, analogous to Fig 4. The dashed line indicates the country-level mean *G_e_*. Panel (b) shows a map of China where the provinces are color-coded according to the inhomogeneity index *G_e_*. It can be seen that the development dependence as found in Fig 4 does also hold on the sub-national scale—at least in China. Provinces with lowest and highest *G_e_*-values are Hong Kong and Tibet, respectively. Note, however, that for the USA we do not find sub-national correlations (S1 Fig in S1 File). (Data source of China level-1 administrative boundaries: https://www.naturalearthdata.com/downloads/10m-cultural-vectors/10m-admin-1-states-provinces/).

Next we analyzed whether the correlations between *G_e_* (for ODIAC) and GDP per capita among countries also appear within a country. Taking China as an example, we disaggerate the national data into provinces. Analogously as for the countries, we calculate cumulative emissions vs. cumulative population and determine the inhomogeneity index at the province level. In Fig 6(a) the *G_e_*-values are plotted vs. the corresponding GDP per capita values, as in Fig 4 but now for provinces. Similar to the country analysis and even more pronounced, we find statistically significant correlations (*ρ* = −0.87, p-value: <0.01).

We performed the corresponding sub-national analysis for the USA on the state level. However, we could not find significant correlations (see S1 Fig in S1 File). Despite this lack of correlations, we find a spatial pattern in the USA. States at the west coast and in the Northeast tend to have larger *G_e_*-values. This is also the case for other states at the east coast and in the Midwest. States in the south as well as Montana, North Dakota, South Dakota tend to have more extreme *G_e_*-values. Repeating the analysis for the Vulcan data, which might be considered the most detailed data, still no correlations between *G_e_* and GDP per capita within the USA are found (S2 Fig in S1 File). However, the analysis does show weak correlations between the *G_e_*-values of Vulcan and ODIAC data. This may imply that, albeit based on a relatively simple disaggregation scheme, the ODIAC datasets are able to describe the spatial inhomogeneity of CO2 emissions at a large scale comparably well as a more complex bottom-up based CO2 emission data, particularly in countries where an accurate CO2 inventory is available.

### Robustness of *G_e_*

**Fig 7:**
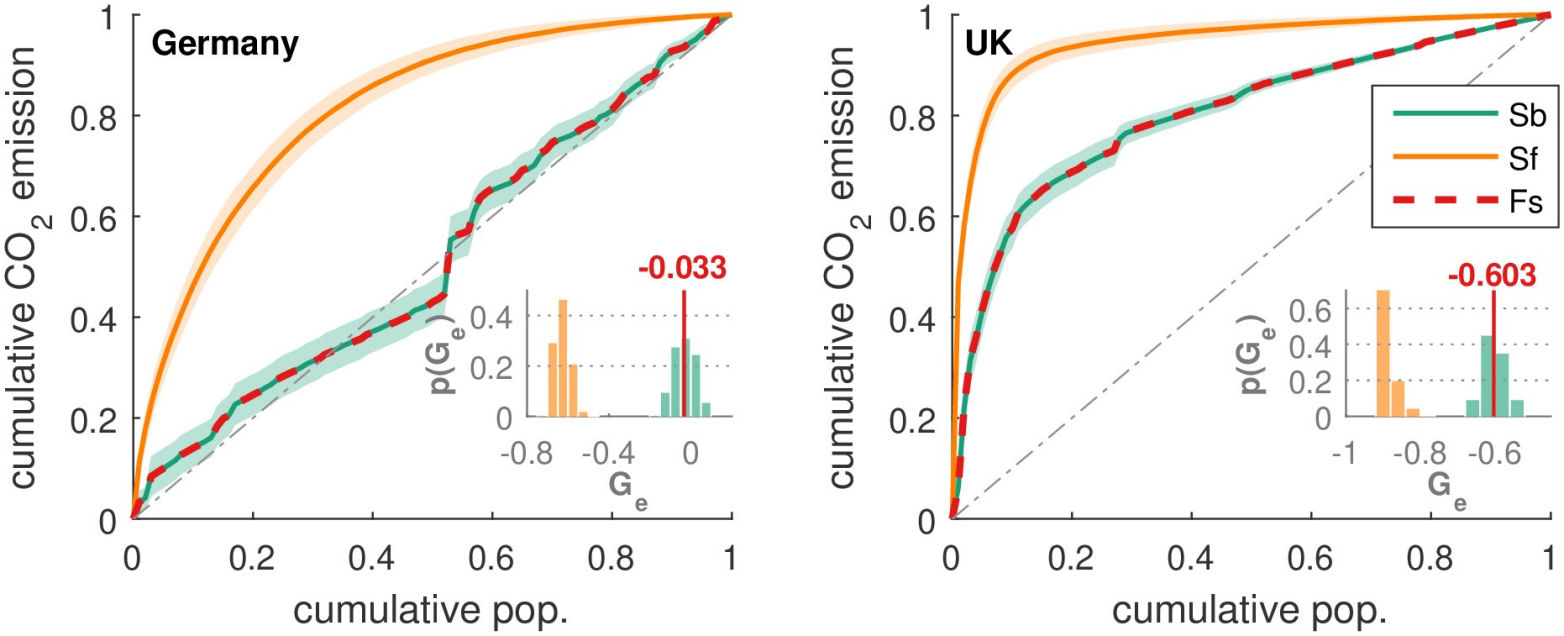
Robustness of *G_e_*. In order to illustrate the robustness of the curves in Fig 3 we compare them with curves when the data is sub-sampled or shuffled. The panels for (a) Germany and (b) UK include the curves for the full samples (Fs), median and envelop for the sub-sampled data (Sb, green), and the median and envelop for the shuffled data (Sf, orange). The insets show histograms of the corresponding inhomogeneity indices. It can be seen that sub-samples of the data lead to similar results as for the full sample so that the results are not due to individual pixels. The *G_e_* values from the shuffling approach −1, as the correlations between population and CO_2_ are destroyed.

Lastly we checked the robustness of the *G_e_* coefficient. We explored different forms of sampling and randomization. In order to check the influence of outliers, we create random sub-samples of the ODIAC data. We constructed a set with 50% of the original size by randomly selecting pairs of population and emissions values from the original set without replacement for 1000 iterations. We calculated the cumulative quantities as before and determined the inhomogeneity index. Repeating the procedure we can assess the statistical spread. As observed in Fig 7, the resampling has minor influence on the shape of the curve and the resulting *G_e_*-values. For Germany, Fig 7(a), 95% of the realizations lead to *G_e_*-values in the range of -0.131 to 0.063, with a median and mean of -0.032, which is very close to the measured value -0.033. The sub-sampled robustness check for the UK led to analogous findings [Fig 7(b)].

Another way to randomize is to shuffle. Since in the analysis we have already sorted the data, we now shuffle only the emissions data and destroy the correlations between emissions and population. Then we perform the whole analysis and obtain cumulative emissions and population curves as well as *G_e_*-values. Repeating the procedure we can assess the statistical spreading. The results are also displayed in Fig 7, and we find that the curves for the shuffled data are very different from the original curves which shows that the actual shapes in Fig 3 are due to the correlations between emissions and population. The shape of the curves for the shuffled data differs between Germany and the UK, Fig 7(a) and 7(b). Since shuffling destroys any correlations, the actual form of the curves can be attributed to a combination of the probability distributions of the population and emissions which differ among the countries.

### Relation to urban scaling

The analysis of CO_2_ efficiency that is carried out here using quasi-Lorenz curves can be related to the urban scaling approach as advocated in [13]. The urban scaling approach aims to establish a parametric relationship between the urban population *P*_u_ of a city and the respective emissions *E*_u_. In our analysis we do not analyze urban population and urban emissions explicitly but examine gridded population *P*_g_ and emission data *E*_g_ within countries. Since urban areas are usually characterized by high population densities (depending on the pixel size), one could transfer the idea of urban scaling to our setting and assume the scaling relationship $E_{g}∼P_{g}^{\beta }$. The case of *β* < 1 indicates CO_2_ efficiency gains with increasing population (density) while *β* > 1 is associated with efficiency losses. Here it is of interest how the non-parametric quasi-Gini coefficient *G_e_* is related to the parametric scaling exponent *β*.

Generally there is no simple association between *β* and *G_e_*. Empirically, the *β* coefficient is usually estimated as the slope of a linear regression of the logarithmic quantities. Hence, it depends on the correlations among the logarithmic quantities cor{log *P*_g_, log *E*_g_} and on the variance of log *P*_g_ and log *E*_g_ only. By contrast, *G_e_* as a non-parametric estimator depends on the exact form of the joint distribution of *P*_g_ and *E*_g_. However, it is possible to determine a specific expression for the relationship between *G_e_* and *β* under certain conditions. The coefficients are related via [35]

$$\begin{array}{c} G_{e}=\frac{\beta -1}{2\lambda -\beta -1}\;, \end{array}$$ (1)

if *P*_g_ is Pareto distributed with shape parameter λ > 1 and a scaling relation of the form $E_{g}∼P_{g}^{\beta }$ with *β* < λ holds *exactly*.

For a detailed derivation see Sec.2 in SI. The formula shows that a scaling coefficient *β* > 1 is associated with *G*_*e*_ > 0. Equivalently, *β* < 1 implies *G*_*e*_ < 0. If $E_{g}∼P_{g}^{\beta }$ holds only approximatively, Eq (1) should still give a reasonable approximation.

Under this scenario, our finding of development dependent *G*_*e*_-values implies a corresponding development dependence of the scaling exponent *β*. Accordingly, in developing countries large cities are typically less emission efficient and vice versa in developed countries.

## Discussion and conclusions

In summary, we have analyzed the correlations between the spatial distribution of population with CO_2_ emissions using high resolution datasets. In order to understand these correlations we employed the quasi-Lorenz curve. The shape of the curve indicates to which extent locations of high or low population emit relatively more or less CO_2_. We characterized the inhomogeneity by a generalized Gini coefficient. For the ODIAC and FFDAS data it depends on the socio-economic development of the considered country (developing countries exhibit relatively more emissions in locations of high population). For the EDGAR data there is no development dependence (overall relatively more emissions in locations of low population). Within China, the development dependence persists for the ODIAC data, but within the USA it vanishes for the ODIAC and Vulcan data. Sub-sampling and shuffling supports the robustness of our analysis.

There is a well-known association between urbanization, economic development, and carbon emissions. However, the quantitative relations behind this association are less understood. Here we show that also the location of emissions is influenced by the economic development. We conclude that during the course of development a spatial separation of emission source and population happens, based on the results for the ODIAC and FFDAS data. This means to some extent high-emitting sources relocate away from locations of large population. A possible explanation could be an increasing environmental consciousness and adoption of cleaner technologies—a trend similar to the environmental Kuznets curve (EKC). Another possibility could be altering composition of economic sectors from agriculture over emission intensive industry to service [36]. While a majority of national mitigation strategies target specific sectors, our results suggest a complementary spatial perspective to prioritize mitigation actions. Depending on the considered scope of emissions, these would be sparsely populated regions in developed countries and densely populated regions in developing and transition countries. Particular attentions should be paid to the latter, as these countries are projected to become more urbanized in the upcoming decades, which entails further rural-urban migration.

The difficulty in explaining the observed phenomenon of country-specific inhomogeneity indices may be attributable to a complex interplay of human activity on local, country, and international scale which entails more evaluation. Concentration or dispersion of human activities is strongly linked to the extent of urban sprawl. Such structural properties certainly affect both the population and the emissions. Moreover, as mentioned earlier, the proxies used to downscale national level CO_2_ emissions and the sectors included while calculating the national level emission data will also impact the spatial inhomogeneity of the origin of CO_2_ emissions. In addition, the location of point sources is an important aspect that can hardly be generalized on the national or even international scale. Maybe, a starting point could be a better understanding of the spatial characteristics of CO_2_ efficiency. Explaining the presented phenomenon—i.e. development dependent concentration of emissions in locations of high or low population—remains a challenge for future research.

Our results for the ODIAC and FFDAS data are consistent with previously reported findings [14], according to which in developing countries large cities are comparably less efficient in terms of CO_2_ emissions, and in developed ones small cities are less efficient. On the one hand, the present study provides stronger empirical evidence, e.g. because it is based on more data and the signatures are more pronounced. On the other hand, the methodology of the present study does not rely on any city definition [37, 38] or any assumption about the functional form of the correlations between population and emissions [39].

We argue that the affirmation “large cities are less green” [11] needs to be revised. According to our results only in developing countries large cities are less green. In developed countries, including the USA, the opposite is the case, relatively more emissions stem from small cities. Anyways, we find it misleading to speak about “green cities” in the context of urban CO_2_ emissions [7], since greenness usually refers to urban vegetation or metaphorically to pollution (while CO_2_ is a colorless gas which as a GHG contributes to global warming).

Certainly, our analysis also has some potential caveats which we want to discuss briefly. The analysis stands and falls with the employed input data, so we cannot exclude to obtain other results if we use other population or emissions data as inputs. Why the EDGAR data leads to different results compared to ODIAC and FFDAS is an interesting problem requiring further research. Moreover, our curves, such as in Fig 3, can have (multiple) crossings with the diagonal, and the index *G_e_* cannot capture to a full extent more complex shapes of the curves.

Another aspect that could be addressed in future studies is the role of the population density [6, 12, 14, 40, 41]. Here we avoid any discussion about city definitions by simply taking gridded data. Since the grid cells are approximately of equal area, the population count and the density are approximately identical. In order to investigate the influence of the density, a suitable city definition—joining grid-cells—will be necessary.

## Supporting information

S1 File (PDF)
