# Supplementary material for: A Gini approach to spatial CO2 emissions
Source: PLoS One. 2020 Nov 18;15(11):e0242479. doi: 10.1371/journal.pone.0242479 (PMC7673567; doi:10.1371/journal.pone.0242479)
Supplement: S1 File — (PDF) [file pone.0242479.s001.pdf]

S1 File

Additional Figures

In S1(a) Fig the G_e-values are plotted vs. the corresponding GDP per capita values, as in Fig 4 in the main text, but here for states in the USA (analogous to Fig 6 in the main text). In contrast to the country analysis, we do not find correlations ($\rho = 0.07$, p-value: 0.64, not statistically significant). However, the G_e-values are consistently in the negative range so that overall high population densities come along with lower CO_2 per capita (consistent with Fig 4 in the main text).

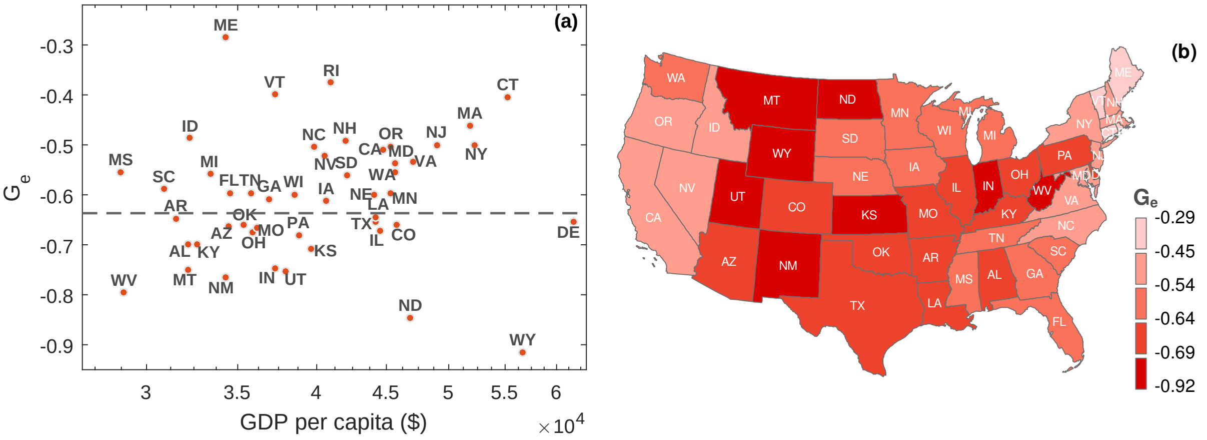

S1 **Fig: Sub-national inhomogeneity index G_e for the USA.** (a) The G_e-values are plotted against the corresponding state GDP per capita values on a logarithmic scale (excluding District of Columbia), analogous to Fig 4 in the main text. The dashed line indicates the country-level mean G_e. (b) Map of contiguous USA where the states are color-coded according to the inhomogeneity index G_e. The development dependence found in Fig 4 does not hold on the sub-national scale – at least for the USA. However, spatially the values are not random: large G_e-values occur at the east and west coasts while smaller ones occur in the predominantly sparsely populated states.

Results of the analogous analysis for the USA and the Vulcan data are displayed in S2(a) Fig. As can be seen, still there are no correlations between the obtained G_e-values and the GDP per capita. Comparing the resulting G_e-values from the Vulcan data with those obtained for the ODIAC data, we do find weak correlations [S2(b) Fig]. In comparison to the ODIAC, the Vulcan data

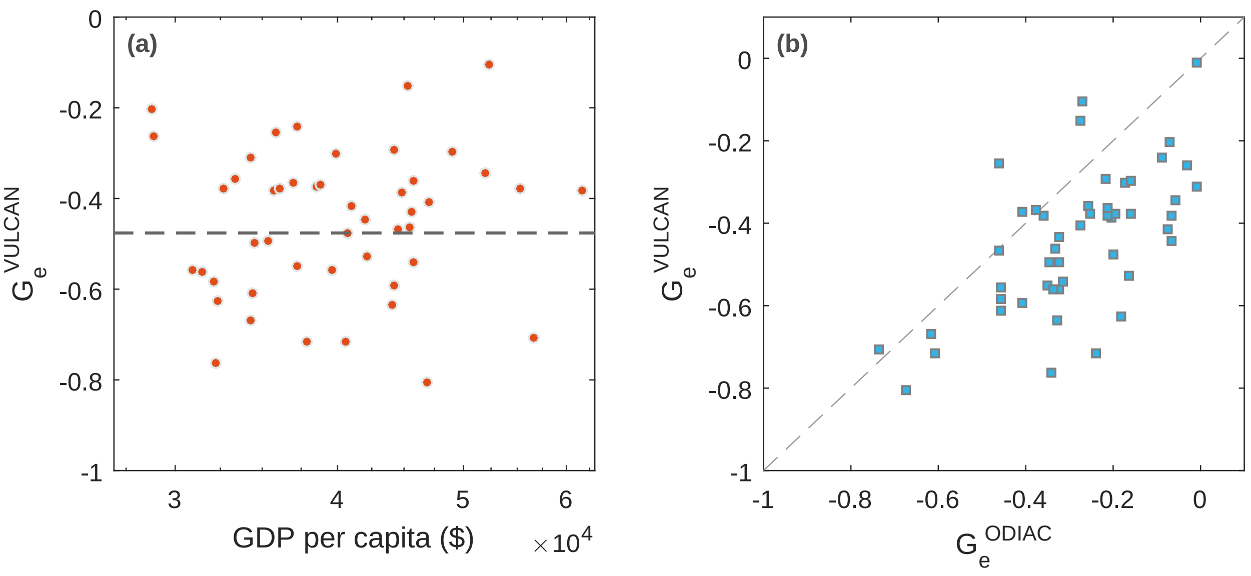

S2 **Fig: Sub-national inhomogeneity index G_e based on the Vulcan data.** We calculated the G_e on the state level for the USA based on th Vulcan data for the year 2002 at 10 km resolution[1,2] . In (a) the G_e-values are plotted against the corresponding state GDP per capita values on a logarithmic scale (excluding District of Columbia), analogous to Fig 1(a). The dashed line indicates the country-level mean G_e. It can be seen that also for the Vulcan data the development dependence does not hold on the sub-national scale in the USA. In (b) we show the correlations between the G_e obtained from the ODIAC data and the corresponding values obtained from the Vulcan data.

overall tends to exhibit lower G_e-values, indicating that there are more emissions from sites of low population.

S1 **Appendix: Derivation of the relationship between β and G_e**

Denoting probability distribution functions with F, the theoretical quasi-Lorenz curve for emissions $E \sim F_E$ with respect to population $P \sim F_P$ is defined as

$$L_{E\circ P}(\theta) = \frac{1}{\mu_E} \int_{-\infty}^{S_P^{-1}(\theta)} \mu_{E|P}(t)\mathrm{d}F_P(t) \quad 0 \leq \theta \leq 1 \tag{S1}$$

where μ_P and μ_E are the respective means of P and E and $\mu_{E|P}$ is the conditional mean of E given P. In contrast to the classical concentration curves[3], the upper boundary of integration is given through the generalized inverse of $S_P(p)$

$$S_P^{-1}(\theta) = \inf\{p : S_P(p) \geq \theta\}. \tag{S2}$$

We call $S_P(p)$ the share function defined as

$$S_P(p) = \frac{1}{\mu_P} \int\limits_{-\infty}^{p} t\mathrm{d}F_P(t). \tag{S3}$$

If we assume that the population P is Pareto distributed with shape parameter $\lambda > 1$ and scale $p_{\min} > 0$, the inverse share function $S_P^{-1}(\theta)$ is given through

$$S_P^{-1}(\theta) = p_{\min}(1-\theta)^{\frac{1}{1-\lambda}}. \tag{S4}$$

If we further assume that the scaling relation $E = aP^\beta$ holds, the conditional mean is simply given as $\mu_{E|P}(t) = at^\beta$ and the unconditional mean for $\beta < \lambda$ can be calculated as

$$\mu_E = \frac{\lambda}{\lambda-\beta} a p_{\min}^\beta. \tag{S5}$$

If $\beta \geq \lambda$ the unconditional mean becomes infinite and the quasi-Lorenz curve can not be computed. Given the previous assumptions the quasi-Lorenz curve can be derived as

$$L_{E\circ P}(\theta) = \left[\frac{\lambda}{\lambda-\beta} a p_{\min}^\beta\right]^{-1} \int\limits_{p_{\min}}^{p_{\min}(1-\theta)^{\frac{1}{1-\lambda}}} a\lambda p_{\min}^\lambda t^{\beta-\lambda-1}\mathrm{d}t \tag{S6}$$

which simplifies to

$$L_{E\circ P}(\theta) = 1 - (1-\theta)^{\frac{\lambda-\beta}{\lambda-1}}. \tag{S7}$$

The generalized Gini coefficient G_e is then given by

$$G_e = 1 - 2\int\limits_0^1 L_{E\circ P}(\theta)\mathrm{d}\theta = \frac{\beta - 1}{2\lambda - \beta - 1} \tag{S8}$$

as stated in Eq (1) in the main text.

References

[1] Gurney, K. R. *et al.* High Resolution Fossil Fuel Combustion CO 2 Emission Fluxes for the United States. *Environmental Science & Technology* **43**, 5535–5541 (2009).

[2] Gurney, K. R. *et al.* Quantification of fossil fuel CO2 emissions on the building/street scale for a large U.S. city. *Environmental science & technology* **46**, 12194–202 (2012).

[3] Yitzhaki, S. & Olkin, I. *Concentration indices and concentration curves*, vol. 19 of *Lecture Notes–Monograph Series*, 380–392 (Institute of Mathematical Statistics, 1991). URL https://doi.org/10.1214/lnms/1215459867.
